# Changes in alcohol consumption after a natural disaster: a study of Norwegian survivors after the 2004 Southeast Asia tsunami

**DOI:** 10.1186/1471-2458-13-58

**Published:** 2013-01-22

**Authors:** Astri Nordløkken, Hilde Pape, Tore Wentzel-Larsen, Trond Heir

**Affiliations:** 1 Norwegian Center for Violence and Traumatic Stress Studies, Kirkeveien 166, House no. 48, Oslo, NO, 0450, Norway; 2 Norwegian Institute for Alcohol and Drug Research, PB 565 Sentrum, Oslo, 0105, Norway; 3 Centre for Child and Adolescent Mental Health, Eastern and Southern Norway, Gullhaug Torg 4B, Oslo, 0484, Norway

**Keywords:** Alcohol use, Disasters, Post-traumatic stress, Epidemiology

## Abstract

**Background:**

Many studies suggest that disaster exposure is related to a subsequent increase in alcohol consumption. Most of these studies have relied on retrospective self-reports to measure changes in alcohol use. The aim of the present study was to examine the association between disaster exposure and drinking behaviors more closely, analyzing data on both self-perceived changes in alcohol consumption and current drinking habits in groups with different extents of disaster exposure.

**Methods:**

A sample of Norwegian adults (≥ 18 years) who resided in areas affected by the 2004 Southeast Asia tsunami (N = 899) were assessed by a postal questionnaire 6 months after the disaster. Based on detailed questions about experiences with the tsunami, participants were grouped according to their extent of disaster exposure. The Impact of Event Scale-Revised was applied to measure the level of post-traumatic stress. Participants were asked whether they had increased or decreased their alcohol consumption after the disaster. Moreover, weekly alcohol consumption and frequency of intoxication during the past month were used as indicators of current drinking behaviors.

**Results:**

Severely exposed individuals more often reported changing their alcohol consumption compared with those who were less exposed. Severe exposure to the tsunami was associated with both a self-perceived increase (OR 21.38, 95% CI 2.91–157.28) and decrease in alcohol consumption (OR 7.41, 95% CI 1.74–31.51). The odds ratios decreased and were not significant when adjusting for post-traumatic stress symptoms. Weekly consumption and frequency of intoxication during the past month did not vary with extent of disaster exposure.

**Conclusions:**

Our findings indicate a polarization effect of severe disaster exposure on self-perceived changes in alcohol consumption; that is, disaster exposure was associated with self-perceived increases and decreases in drinking. However, the absence of associations between disaster exposure and indicators of current drinking behaviors suggests that the observed polarization effect may be overestimated because of attribution and recall bias.

## Background

Numerous studies have investigated how exposure to trauma and post-traumatic stress symptoms are associated with the use and abuse of alcohol [1-7]. Most of this research has focused on the extent of comorbidity between post-traumatic stress disorder (PTSD) and diagnosis of alcohol abuse or dependence. Some recent studies investigated whether alcohol consumption increases among individuals exposed to trauma. According to a recent review [8], a positive relationship between trauma exposure and increased alcohol consumption has been documented for accidents [9,10], terrorist events [11-14], and natural disasters [15-17]. In some of these studies the trauma exposure refers to the presence of PTSD-symptoms, in others to trauma exposure more generally. For example, studies conducted in New York after the 9/11 terrorist attacks found that 25% of citizens living proximal to the World Trade Center reported a self-perceived increase in alcohol use 5–8 weeks after the attacks [18], and that 17% still reported increased use after 6 months [19]. Literature suggesting that alcohol is a way of self-medicating PTSD makes this relationship between trauma exposure and increased drinking plausible [3,7,20].

Whether trauma exposure and post-traumatic stress are also related to reduced drinking has rarely been investigated. However, drinking typically occurs in the company of other people, and there is evidence that some trauma-exposed individuals tend to withdraw from social life [21,22]. Hence, one may assume that trauma exposure might also be associated with a subsequent tendency to drink less. Indeed, two studies documented self-perceived reductions in alcohol consumption in certain individuals after exposure to trauma [7,23]. The present study adds to this meager literature by investigating both increased and decreased alcohol consumption among survivors after a natural disaster.

Evidence suggesting that exposure to disaster increases the risk of more extensive drinking is important with respect to treatment and prevention, and several authors have called for more research in this field [15,19]. An expedient design for investigating the issue is a prospective study comparing pre-disaster and post-disaster alcohol use, but a lack of pre-disaster data is a common limitation in this research literature. Most of the research on associations between disaster exposure and subsequent alcohol consumption has used retrospective reports of self-perceived changes in alcohol consumption. A crucial limitation of this approach is the possibility of attribution and recall bias [24]. However, the impact of such limitations has not yet been documented for studies of the relationship between disaster exposure and alcohol consumption.

Data from a sample of Norwegians who were in Southeast Asia during the 2004 tsunami offered a unique opportunity for investigating the relationship between disaster exposure and alcohol consumption. Geographic differences in disaster severity resulted in a target group that was directly exposed to the tsunami as well as a reference group that was not, enabling us to compare alcohol consumption in individuals with different extents of disaster exposure.

The purpose of this study was to investigate more closely the association between the extent of disaster exposure and drinking behaviors. More specifically, we aimed to investigate the association between disaster exposure and alcohol consumption using two different approaches: retrospective reports of self-perceived changes in alcohol consumption, and indicators of current drinking behaviors in groups with different extents of disaster exposure.

## Methods

### Sample and data collection

The study population comprised Norwegians aged 18 years or older who were repatriated from disaster-stricken areas after the 2004 tsunami [25]. The vast majority of participants (96%) were in Southeast Asia on vacation at the time of the disaster. This sample received a postal questionnaire 6 months after the disaster (response rate 36%, N = 899). However, our analyses were confined to 883 individuals because of missing data on exposure. The respondents were of similar age, but a greater proportion were women compared with non-responders [21]. Attrition was negatively associated with exposure to the tsunami and the severity of post-traumatic stress symptoms [26]. Employment status and marital status of participants were similar to the age- and sex-adjusted Norwegian population [27]. The study was approved by the Regional Committee for Medical Research Ethics and Norwegian Social Science Data Services.

### Measures

#### Disaster exposure

Participants were asked in detail about their exposure to stressful aspects of the tsunami [28] and classified into three groups according to exposure severity based on these responses [21,25]: not exposed, non-danger exposed, and danger exposed. The ‘not exposed’ group included individuals who reported no contact with the waves or flood, no physical injuries to themselves or a close relative, no loss of a relative, no fear for the safety of relatives, and no witnessing of death or suffering of others. The ‘non-danger exposed’ group included individuals with some disaster-related exposure but no life-threatening experiences. The ‘danger exposed’ group included individuals who had life-threatening experiences, such as having been caught, touched, or chased by the waves or flood.

#### Alcohol consumption

Self-perceived changes in alcohol consumption were assessed by asking the participants whether they changed their alcohol consumption after the tsunami, using a response scale ranging from 1 (‘drink much more’) to 5 (‘drink much less’). As a result of low frequencies in the first and last categories, the item was trichotomized into ‘increased drinking’ , ‘unchanged’ , and ‘decreased drinking’. Dichotomized versions of this variable (increased drinking vs. not increased, and decreased drinking vs. not decreased) were also used. The participants were also asked how many alcoholic drinks they consumed in a typical week (a ‘drink’ was specified as a glass of wine, a beer, or a mixed drink, i.e. similar to a standard unit of alcohol). Response categories were Do not drink alcohol, 1 drink, 2–5 drinks, 6–10 drinks, 11–20 drinks, and > 20 drinks. Last, heavy episodic drinking was assessed by asking the participants how many times during the last month they had consumed so much alcohol that they felt clearly intoxicated. Response categories were None, Once, 2–3 times, 4–10 times, and > 10 times.

#### Post-traumatic stress symptoms

The Impact of Event Scale-Revised (IES-R) [29] was applied to measure the intensity of post-traumatic stress symptoms during the previous week with regard to the participants’ experience with the tsunami. The IES-R comprises 22 items measuring symptoms of intrusion, avoidance, and arousal. The response scale ranges from 0 (not at all) to 4 (extremely). Previous research has demonstrated high scale-construct validity and test-retest reliability of this inventory [30,31], and a high internal consistency was revealed in the present study (Cronbach alpha = 0.96). The mean IES-R score was calculated as a measure of the severity of post-traumatic stress symptoms and assigned a missing value for individuals with seven or more unanswered items. Otherwise, the scale score was computed as the mean of the valid items.

### Statistical analysis

We performed exact chi-square tests using Monte Carlo simulation with 100,000 sampled tables when necessary, or one-way ANOVAs, with subsidiary Holm-corrected post-hoc tests to compare the three allocated groups with regard to demographic variables, post-traumatic stress, and alcohol consumption. Initially, sex-specific subgroup analyses of the associations between disaster exposure and alcohol use were performed. Because sex-specific cell frequencies were rather small, and the association between disaster exposure and alcohol use was similar for men and women, we chose to report total sample results only. We applied logistic regression models to analyze associations between exposure and self-perceived changes in alcohol consumption, using increased drinking (versus not increased) and decreased drinking (versus not decreased) as dependent variables. In bivariate analyses, demographic variables, the extent of disaster exposure, and mean IES-R score were introduced as independent variables. Based on the number of individuals in the smallest group, we decided to include independent variables, resulting in a total of 5 degrees of freedom in the multivariate models [32]. In addition to disaster exposure, we added age and sex, and then the mean IES-R score, as independent variables in hierarchical regression analyses. Significance was set as p < 0.05. Statistical analyses were performed using the software package SPSS version 19.0.

## Results

### Allocated reference and exposure groups

Table 1 provides the distribution of age, sex, education level, employment status, and marital status in the allocated reference and exposure groups. The groups differed significantly with respect to sex and age, but there were no significant variations in education level, employment status, or marital status. The average IES-R score varied with the level of exposure, from 0.39 (SD = 0.47) in the not exposed reference group to 1.07 (SD = 0.80) in the non-danger exposed group and 1.47 (SD = 0.80) in the danger exposed group (all pairwise p-values < 0.001).

**Table 1:** Demographic variables in the reference and exposure groups

	**Total sample**	**Not exposed, reference**	**Non-danger exposed**	**Danger exposed**
	**(N = 883)**	**(n = 149, 16.9%)**	**(n = 444, 50.3%)**	**(n = 290, 32.8%)**
**Age, years**				
Mean (SD)	43.6 (12.9)	46.9^1^ (12.6)	42.7^1^ (12.6)	43.5^1^ (13.4)
Range	18–82	21–82	18–79	18–78
**Sex, n (%)**				
Female	465 (52.7)	65 (43.6)^2^	246 (55.4)^2^	154 (53.1)^2^
Male	418 (47.3)	84 (56.4)^2^	198 (44.6)^2^	136 (46.9)^2^
**Education, n (%)**				
≤ 12 years	328 (40.4)	49 (37.4)	172 (41.7)	107 (39.8)
> 12 years	484 (59.6)	82 (62.6)	240 (58.3)	162 (60.2)
**Employed, n (%)**				
Yes	652 (73.8)	114 (76.5)	323 (72.7)	215 (74.1)
No	231 (26.2)	35 (23.5)	121 (27.3)	75 (25.9)
**Married or cohabiting, n (%)**				
Yes	583 (70.0)	94 (70.7)	295 (69.6)	194 (70.3)
No	250 (30.0)	39 (29.3)	129 (30.4)	82 (29.7)

Tests and values are only reported when a significant difference was found.

^1^ANOVA: F(df = 2, n = 872) = 5.78, p = 0.003, η^2^ = 0.01.

^2^Chi-square test: χ^2^ = 6.25, p = 0.044.

### Self-perceived changes in alcohol consumption

Self-perceived changes in alcohol consumption after the disaster differed between the three groups (Table 2). The danger exposed group more often reported *increased* drinking compared with the non-danger exposed group (χ^2^ = 7.48, p = 0.019), and the non-danger exposed group more often reported increased drinking compared with the reference group (χ^2^ = 8.70, p = 0.010). Furthermore, the danger exposed group more often reported *decreased* drinking compared with the non-danger exposed group (χ^2^ = 6.15, p = 0.026) and the reference group (χ^2^ = 9.91, p = 0.005).

**Table 2:** Associations between disaster exposure and self-perceived changes in alcohol consumption

	**Total sample**	**Not exposed, reference**	**Non-danger exposed**	**Danger exposed**
	**(N = 830)**	**(n = 123)**	**(n = 432)**	**(n = 275)**
Increased drinking	78 (9.4)	1 (0.8)	36 (8.3)	41 (14.9)
Unchanged	695 (83.7)	120 (97.6)	371 (85.9)	204 (74.2)
Decreased drinking	57 (6.9)	2 (1.6)	25 (5.8)	30 (10.9)
χ^2^ = 37.29, p < 0.001				

Results are given as N (% within group).

A self-perceived *increase* in alcohol consumption was bivariately associated with male sex, not being married or cohabiting, the extent of disaster exposure, and the severity of post-traumatic stress symptoms (Table 3). A self-perceived *decrease* in alcohol consumption was bivariately associated with younger age, being in the danger exposed group, and the severity of post-traumatic stress symptoms.

**Table 3:** Bivariate associations between self-perceived changes in alcohol consumption and demographic variables, disaster exposure, and post-traumatic stress symptoms

		**Increased drinking**		**Decreased drinking**	
		**(N = 830)**		**(N = 830)**	
	**Total (N)**	**N (%) or mean**	**OR (95% CI)**	**N (%) or mean**	**OR (95% CI)**
**Age**^**a,b**^	830	43.4 vs. 43.4	1.00 (0.83–1.20)	38.7 vs. 43.8	0.72** (0.58–0.90)
**Sex**					
Female	440	31 (7.0)	-	33 (7.5)	-
Male	390	47 (12.1)	1.81* (1.12–2.91)	24 (6.2)	0.81 (0.47–1.39)
**Education**					
≤ 12 years	305	34 (11.1)	-	19 (6.2)	-
> 12 years	470	40 (8.5)	0.74 (0.46–1.20)	34 (7.2)	1.17 (0.66–2.10)
**Married or cohabiting**^**a**^					
Yes	557	44 (7.9)	-	36 (6.5)	-
No	239	33 (13.8)	1.87* (1.16–3.02)	20 (8.4)	1.32 (0.75–2.34)
**Employed**^**a**^					
Yes	627	61 (9.7)	-	37 (5.9)	-
No	203	17 (8.4)	0.85 (0.48–1.49)	20 (9.9)	1.74 (0.99–3.08)
**Disaster exposure**					
Not exposed	123	1 (0.8)	-	2 (1.6)	-
Non-danger exposed	432	36 (8.3)	11.09* (1.51–81.73)	25 (5.8)	3.72 (0.87–15.91)
Danger exposed	275	41 (14.9)	21.38** (2.91–157.28)	30 (10.9)	7.41** (1.74–31.51)
**IES-R**^**c**^**(mean score)**	815	1.80 vs. 1.02	2.88*** (2.17–3.82)	1.52 vs. 1.06	1.84*** (1.36–2.49)

* p-value < 0.05.

** p-value < 0.01.

*** p-value < 0.001.

^a^ At the time of the disaster.

^b^ OR per 10 years.

^c^ Impact of Event Scale-Revised.

The association between disaster exposure and self-reported changes in drinking habits remained significant when adjusting for age and sex (Table 4). When adjusting for post-traumatic stress symptoms, the odds ratios decreased and were no longer significant.

**Table 4:** Multivariate associations between self-perceived changes in alcohol consumption and disaster exposure

	**Increased drinking**	**Decreased drinking**
	**(N = 830)**	**(N = 830)**
	**OR (95% CI)**	**OR (95% CI)**
**Step 1: Unadjusted**		
Not exposed (reference)	-	-
Non-danger exposed	11.09* (1.51–81.73)	3.72 (0.87–15.91)
Danger exposed	21.38** (2.91–157.28)	7.41** (1.74–31.51)
**Step 2: Adjusted for age**^**a**^** and sex**		
Not exposed (reference)	-	-
Non-danger exposed	11.94* (1.62–88.26)	3.30 (0.77–14.20)
Danger exposed	22.92** (3.11–169.07)	6.69* (1.57–28.59)
**Step 3: Adjusted for age, sex, and IES-R**^**b**^** mean score**		
Not exposed (reference)	-	-
Non-danger exposed	5.00 (0.66–37.96)	2.18 (0.49–9.67)
Danger exposed	7.03 (0.92–53.81)	3.69 (0.82–16.63)

* p-value < 0.05.

** p-value < 0.01.

^a^ At the time of the disaster.

^b^ Impact of Event Scale-Revised.

### Current drinking behaviors

Based on the above findings, one would expect the proportion of abstainers or very light drinkers, as well as the proportion of heavy drinkers, to be elevated in the severely exposed group. However, analyses of the two measures on current drinking behaviors indicated that this was not the case. Neither weekly consumption of alcohol (Table 5) nor frequency of intoxication during the past month (Table 6) varied significantly between the reference and exposure groups, either in the total sample or in men and women separately. We performed post-hoc analyses of high consumers (consumption of > 10 units of alcohol weekly) in the allocated groups. The proportion of high consumers did not differ significantly between the three exposure groups (χ^2^ = 3.03, p = 0.219).

**Table 5:** Associations between disaster exposure and weekly alcohol consumption (number of units consumed)

	**Total sample**	**Not exposed, reference**	**Non-danger exposed**	**Danger exposed**
	**(N = 840)**	**(n = 122)**	**(n = 434)**	**(n = 284)**
None	76 (9.0)	8 (6.6)	35 (8.1)	33 (11.6)
1 unit	211 (25.1)	41 (33.6)	107 (24.7)	63 (22.2)
2–5 units	320 (38.1)	46 (37.7)	168 (38.7)	106 (37.3)
6–10 units	171 (20.4)	18 (14.8)	86 (19.8)	67 (23.6)
11–20 units	43 (5.1)	7 (5.7)	25 (5.8)	11 (3.9)
> 20 units	19 (2.3)	2 (1.6)	13 (3.0)	4 (1.4)
χ^2^ = 14.84, p = 0.134				

Results are given as N (% within group).

**Table 6:** Associations between disaster exposure and frequency of intoxication in the past month

	**Total sample**	**Not exposed, reference**	**Non-danger exposed**	**Danger exposed**
	**(N = 837)**	**(n = 123)**	**(n = 434)**	**(n = 280)**
None	359 (42.9)	57 (46.3)	183 (42.2)	119 (42.5)
Once	212 (25.3)	31 (25.2)	110 (25.3)	71 (25.4)
2–3 times	190 (22.7)	27 (22.0)	98 (22.6)	65 (23.2)
4–10 times	60 (7.2)	7 (5.7)	36 (8.3)	17 (6.1)
> 10 times	16 (1.9)	1 (0.8)	7 (1.6)	8 (2.9)
χ^2^ = 4.37, p = 0.825				

Results are given as N (% within group).

## Discussion

In this study, we found an association between exposure to stressful aspects of a natural disaster and self-perceived changes in alcohol consumption. Individuals who were most severely exposed to the 2004 Southeast Asia tsunami were more likely to report changes in alcohol consumption compared with individuals who were not exposed, suggesting a polarization effect of disaster exposure. However, these findings were not supported by analyses of data on current drinking behaviors. More specifically, disaster exposure was not associated with either the weekly level of alcohol consumption or with the frequency of intoxication during the past month.

The positive association between disaster exposure and a self-perceived increase in alcohol consumption is in agreement with previous research [7,9,12,13,15,18,23]. The relationship between disaster exposure and self-perceived reduction in alcohol consumption has been investigated far less often, but two studies reported reduced drinking in some individuals after disasters [7,23].

The polarization effect of disaster exposure has plausible explanations. Patients with PTSD and alcohol use disorders often report using alcohol to cope with post-traumatic stress [3,7,20], suggesting that alcohol may serve a self-medicating function. On the other hand, reduced drinking might reflect an effort to preserve emotional stability [7,23]. In addition, alcohol consumption is often part of one’s social life [33,34], which may be affected by social withdrawal in the aftermath of a disaster [21,22].

The odds ratios for the associations between disaster exposure and self-perceived changes in alcohol consumption decreased and were no longer significant when we adjusted for the level of post-traumatic stress. Thus, post-traumatic stress may be more important for self-perceived changes in alcohol consumption than the disaster exposure itself [15].

Our finding that current drinking behaviors did not differ significantly between the reference and exposure groups is important when interpreting the association between disaster exposure and self-perceived changes in alcohol consumption. The possibility of attribution and recall bias should be seriously considered. Remembering may depend on the perceived significance of potentially traumatic life events in terms of a person’s identity and whether such events are assigned a central role in one’s life history [35]. Thus, individuals exposed to a disaster, especially those who struggle with post-traumatic stress, may be more likely to attribute lifestyle and behavioral habits to the disaster event.

Questioning the conclusions drawn from reports of self-perceived changes in alcohol consumption is also supported by findings from studies that investigated the relationship between disaster exposure and changes in alcohol consumption using alternative methodology. In a prospective study of individuals living in areas affected by hurricanes Katrina and Rita, alcohol consumption did not increase after the disaster, except among individuals who had a high level of previous lifetime trauma [17]. In a cross-sectional study of disaster relief workers after the 9/11 terrorist attacks, the amount of alcohol consumed was not related to post-traumatic stress [23]. In addition, our finding that the prevalence of high consumers did not differ between reference and exposure groups corresponds with the notion that mass trauma events do not increase the prevalence of alcohol use disorders per se [36].

### Methodological considerations

Data on self-perceived changes in alcohol consumption in the aftermath of a disaster may be affected by cognitive biases, and hence produce unreliable findings. Therefore, the additional assessment of both weekly alcohol consumption and frequency of intoxication during the past month strengthens this study. It is true that the former measure may be called into question because no timeframe was specified, but the item appeared in the context of other questions referring to incidents and events during the previous month.

All Norwegian adults who were in Southeast Asia during the tsunami were asked to participate in the study, reducing sample-selection bias. However, the response rate was moderate, and attrition was inversely related to disaster exposure and post-traumatic stress symptoms [26]. Whether there was also an association between non-participation and alcohol use is unknown. It is true that some studies indicate that there is a positive correlation [37-39], but the research on non-response bias in relation to drinking is meager, and the findings have been mixed [40]. However, the self-selection bias in this study may have been less likely to have influenced the associations we were studying. Systematic response rate biases are more likely to affect frequency estimates of disaster exposure or alcohol consumption than estimates of differences in alcohol consumption between reference and exposure groups [41].

The generalizability of our findings may be questioned for various reasons. The study was based on a sample from a high-income European country with an established welfare system. In addition, the respondents had been exposed to a single, acute disaster and were repatriated shortly after the trauma. Therefore, one may assume that most of them were, to a large extent, shielded from secondary disaster stressors such as economic loss, relocation, and disruption to normal life. Moreover, individuals’ post-traumatic reactions might vary with the nature of the traumatic event, implying that the results may not apply to disasters or traumatic events of a different kind. Finally, it should be noted that the predominant drinking pattern in Norway is characterized by infrequent drinking and a high intake of alcohol when drinking occurs [34]. Thus, the findings cannot be readily generalized to populations with a different consumption pattern.

## Conclusions

Our findings indicate a polarization effect of disaster exposure on self-perceived changes in alcohol consumption. Severely exposed individuals more often reported either increased or decreased drinking. Both associations may be mediated by post-traumatic stress. However, these associations were not reproduced when analyzing other measures of drinking behavior, i.e., we found no significant association between disaster exposure and current alcohol use. This contradiction suggests that the possibility of attribution and recall bias should be seriously considered when interpreting self-reported changes in alcohol consumption after a disaster. Thus, the relationship between disaster exposure and change in alcohol consumption, which has been documented in numerous studies, might be overestimated.

## Competing interests

The authors declare that they have no competing interests.

## Authors’ contributions

AN performed the statistical analyses and drafted the manuscript. HP and TWL contributed to analyzing and interpreting the data. TH participated in developing and carrying out the survey and provided substantial contributions to interpreting the data and writing the manuscript. All authors contributed to critically revising the manuscript, and read and approved the final version.

## Pre-publication history

The pre-publication history for this paper can be accessed here:

http://www.biomedcentral.com/1471-2458/13/58/prepub

## References

[B1] Jacobsen LK Southwick SM Kosten TR Substance use disorders in patients with posttraumatic stress disorder: a review of the literature Am J Psychiatry 2001 158 8 1184 1190 10.1176/appi.ajp.158.8.1184 11481147

[B2] Breslau N Davis GC Schultz LR Posttraumatic stress disorder and the incidence of nicotine, alcohol, and other drug disorders in persons who have experienced trauma Arch Gen Psychiatry 2003 60 3 289 294 10.1001/archpsyc.60.3.289 12622662

[B3] Chilcoat HD Breslau N Posttraumatic stress disorder and drug disorders: testing causal pathways Arch Gen Psychiatry 1998 55 10 913 917 10.1001/archpsyc.55.10.913 9783562

[B4] Stewart SH Alcohol abuse in individuals exposed to trauma: a critical review Psychol Bull 1996 120 1 83 112 8711018 10.1037/0033-2909.120.1.83

[B5] McCarthy E Petrakis I Epidemiology and management of alcohol dependence in individuals with post-traumatic stress disorder CNS Drugs 2010 24 12 997 1007 10.2165/11539710-000000000-00000 21090836

[B6] Kessler RC Sonnega A Bromet E Hughes M Nelson CB Posttraumatic stress disorder in the national comorbidity survey Arch Gen Psychiatry 1995 52 12 1048 1060 10.1001/archpsyc.1995.03950240066012 7492257

[B7] McFarlane A Epidemiological evidence about the relationship between PTSD and alcohol abuse: the nature of the association Addict Behav 1998 23 6 813 826 10.1016/S0306-4603(98)00098-7 9801718

[B8] Keyes KM Hatzenbuehler ML Hasin DS Stressful life experiences, alcohol consumption, and alcohol use disorders: the epidemiologic evidence for four main types of stressors Psychopharmacology 2011 218 1 1 17 10.1007/s00213-011-2236-1 21373787 PMC3755727

[B9] Joseph S Yule W Williams R Hodgkinson P Increased substance use in survivors of the herald of free enterprise disaster Br J Med Psychol 1993 66 Pt 2 185 191 8353111 10.1111/j.2044-8341.1993.tb01740.x

[B10] Kasl SV Chisholm RF Eskenazi B The impact of the accident at the three mile island on the behavior and well-being of nuclear workers; part I: perceptions and evaluations, behavioral responses, and work-related attitudes and feelings Am J Public Health 1981 71 5 472 483 10.2105/AJPH.71.5.472 7212135 PMC1619731

[B11] DiMaggio C Galea S Li G Substance use and misuse in the aftermath of terrorism. A Bayesian meta-analysis Addiction 2009 104 6 894 904 10.1111/j.1360-0443.2009.02526.x 19392912

[B12] Grieger TA Fullerton CS Ursano RJ Posttraumatic stress disorder, alcohol use, and perceived safety after the terrorist attack on the pentagon Psychiatr Serv 2003 54 10 1380 1382 10.1176/appi.ps.54.10.1380 14557524

[B13] Pfefferbaum B Doughty DE Increased alcohol use in a treatment sample of Oklahoma City bombing victims Psychiatry 2001 64 4 296 303 11822207 10.1521/psyc.64.4.296.18598

[B14] Schiff M Benbenishty R McKay M DeVoe E Liu X Hasin D Exposure to terrorism and israeli Youths’ psychological distress and alcohol Use: an exploratory study Am J Addict 2006 15 3 220 226 10.1080/10550490600626200 16923668

[B15] Vetter S Rossegger A Rossler W Bisson JI Endrass J Exposure to the tsunami disaster, PTSD symptoms and increased substance use - an internet based survey of male and female residents of switzerland BMC Publ Health 2008 8 92 10.1186/1471-2458-8-92 PMC2277388 18366682

[B16] Kishore V Theall KP Robinson W Pichon J Scribner R Roberson E Johnson S Resource loss, coping, alcohol use, and posttraumatic stress symptoms among survivors of hurricane katrina: a cross-sectional study Am J Disaster Med 2008 3 6 345 357 19202888

[B17] Cerda M Tracy M Galea S A prospective population based study of changes in alcohol use and binge drinking after a mass traumatic event Drug Alcohol Depend 2011 115 1–2 1 8 20977977 10.1016/j.drugalcdep.2010.09.011 PMC3039709

[B18] Vlahov D Galea S Resnick H Ahern J Boscarino JA Bucuvalas M Gold J Kilpatrick D Increased use of cigarettes, alcohol, and marijuana among manhattan, New york, residents after the september 11th terrorist attacks Am J Epidemiol 2002 155 11 988 996 10.1093/aje/155.11.988 12034577

[B19] Vlahov D Galea S Ahern J Resnick H Boscarino JA Gold J Bucuvalas M Kilpatrick D Consumption of cigarettes, alcohol, and marijuana among New York city residents six months after the september 11 terrorist attacks Am J Drug Alcohol Abuse 2004 30 2 385 407 10.1081/ADA-120037384 15230082

[B20] Stewart SH Pihl RO Conrod PJ Dongier M Functional associations among trauma, PTSD, and substance-related disorders Addict Behav 1998 23 6 797 812 10.1016/S0306-4603(98)00070-7 9801717

[B21] Heir T Sandvik L Weisaeth L Hallmarks of posttraumatic stress: symptom Z-scores in a tsunami-affected tourist population Psychopathology 2009 42 3 157 164 10.1159/000207457 19276641

[B22] Hofmann SG Litz BT Weathers FW Social anxiety, depression, and PTSD in Vietnam veterans J Anxiety Disord 2003 17 5 573 582 10.1016/S0887-6185(02)00227-X 12941367

[B23] Simons JS Gaher RM Jacobs GA Meyer D Johnson-Jimenez E Associations between alcohol Use and PTSD symptoms among american Red cross disaster relief workers responding to the 9/11/2001 attacks Am J Drug Alcohol Abuse 2005 31 2 285 304 15912717

[B24] Aschengrau A Seage GR Essentials of epidemiology in public health 2008 Sudbury: Jones and Bartlett

[B25] Heir T Rosendal S Bergh-Johannesson K Michel PO Mortensen EL Weisaeth L Andersen HS Hultman CM Tsunami-affected Scandinavian tourists: disaster exposure and post-traumatic stress symptoms Nord J Psychiatr 2011 65 1 9 15 10.3109/08039481003786394 20429748

[B26] Hussain A Weisaeth L Heir T Nonresponse to a population-based postdisaster postal questionnaire study J Trauma Stress 2009 22 4 324 328 10.1002/jts.20431 19644976

[B27] Statistics Norway Population, education, workhttp://www.ssb.no/english/

[B28] Heir T Weisaeth L Acute disaster exposure and mental health complaints of Norwegian tsunami survivors six months post disaster Psychiatry 2008 71 3 266 276 18834277 10.1521/psyc.2008.71.3.266

[B29] Wilson JP Keane TM Assessing psychological trauma and PTSD 2004 New York: Guilford Press

[B30] Johansen VA Wahl AK Eilertsen DE Weisaeth L Prevalence and predictors of post-traumatic stress disorder (PTSD) in physically injured victims of non-domestic violence. A longitudinal study Soc Psychiatr Psychiatr Epidemiol 2007 42 7 583 593 10.1007/s00127-007-0205-0 17530151

[B31] Eid J Larsson G Johnsen BH Laberg JC Bartone PT Carlstedt B Psychometric properties of the norwegian impact of event scale-revised in a non-clinical sample Nord J Psychiatr 2009 63 5 1 7 10.1080/08039480903118190 19688636

[B32] Harrell FE Regression modeling strategies: with applications to linear models, logistic regression, and survival analysis 2001 New York: Springer

[B33] Nordlund S What is alcohol abuse? Changes in Norwegians’ perceptions of drinking practices since the 1960s Addiction Res Theor 2008 16 1 85 94 10.1080/16066350701699130

[B34] Horverak Ø Bye EK Det norske drikkemønsteret: en studie basert på intervjudata fra 1973–2004 SIRUS-rapport. vol. nr. 2/2007 2007 Oslo: Statens istitutt for rusmiddelforskning 248s

[B35] Conway MA Memory and the self J Mem Lang 2005 53 594 628 10.1016/j.jml.2005.08.005

[B36] North CS Ringwalt CL Downs D Derzon J Galvin D Postdisaster course of alcohol use disorders in systematically studied survivors of 10 disasters Arch Gen Psychiatry 2011 68 2 173 180 10.1001/archgenpsychiatry.2010.131 20921113

[B37] Zhao J Stockwell T Macdonald S Non-response bias in alcohol and drug population surveys Drug Alcohol Rev 2009 28 6 648 657 10.1111/j.1465-3362.2009.00077.x 19930019

[B38] Lemmens PH Tan ES Knibbe RA Bias due to non-response in a dutch survey on alcohol consumption Br J Addict 1988 83 9 1069 1077 10.1111/j.1360-0443.1988.tb00534.x 3224190

[B39] Knudsen AK Hotopf M Skogen JC Overland S Mykletun A The health status of nonparticipants in a population-based health study: the hordaland health study Am J Epidemiol 2010 172 11 1306 1314 10.1093/aje/kwq257 20843863

[B40] Caetano R Non-response in alcohol and drug surveys: a research topic in need of further attention Addiction 2001 96 11 1541 1545 10.1046/j.1360-0443.2001.961115411.x 11784451

[B41] Søgaard A Selmer R Bjertness E Thelle D The oslo health study: the impact of self-selection in a large, population-based survey Int J Equity Health 2004 3 1 3 10.1186/1475-9276-3-3 15128460 PMC428581

